# Exploring factors influencing implementation across the explanatory-to-pragmatic trial continuum: a sequential qualitative integration of delivering higher-intensity walking exercise within inpatient stroke rehabilitation

**DOI:** 10.1186/s43058-025-00812-y

**Published:** 2026-01-08

**Authors:** Suzanne Ackerley, Stanley H. Hung, Lisa Sheehy, Sarah J. Donkers, Polina Timofeeva, Krista L. Best, Sue Peters, Sarah S. Park, Béatrice Ouellet, Victor E. Ezeugwu, Marie-Hélène Milot, Brodie M. Sakakibara, Janice J. Eng, Louise A. Connell

**Affiliations:** 1 Faculty of Health and Medicine, Health Innovation Campus, Lancaster University, Lancaster, UK; 2 Rakehead Rehabilitation Centre, East Lancashire Hospitals NHS Trust, Burnley General Teaching Hospital, Lancashire, UK; 3 Department of Physical Therapy, University of British Columbia, Vancouver, BC Canada; 4 Centre for Aging SMART, Vancouver Coastal Health Research Institute, Vancouver, BC Canada; 5 Bruyère Health Research Institute, Ottawa, ON Canada; 6 School of Rehabilitation Science, College of Medicine, University of Saskatchewan, Saskatoon, Canada; 7 Centre for interdisciplinary research in rehabilitation and social integration (Cirris), Centre intégré universitaire de santé et de services sociaux de La capitale-nationale, Quebec City, QC Canada; 8 School of Rehabilitation Sciences, Faculty of Medicine, Université Laval, Quebec City, QC Canada; 9 School of Physical Therapy, Faculty of Health Sciences, Western University, London, ON Canada; 10 Gray Centre for Mobility and Activity, Parkwood Institute, London, ON Canada; 11 Department of Physical Therapy, University of Alberta, Edmonton, AB Canada; 12 École de Réadaptation, Faculté de Médecine Et Des Sciences de La Santé, Université de Sherbrooke and Research Center On Aging CIUSSS-de-L’Estrie-CHUS, Quebec, Canada; 13 Department of Occupational Science and Occupational Therapy, University of British Columbia, Vancouver, BC Canada; 14 Centre for Chronic Disease Prevention and Management, University of British Columbia Okanagan, Kelowna, BC Canada

**Keywords:** Intensity, Allied health, Physical therapy, Context, Adaptation, Fidelity, PRECIS-2

## Abstract

**Background:**

Many innovations with proven efficacy in randomized controlled trials encounter significant barriers to real-world implementation. Using an example from delivery of a higher-intensity walking exercise protocol within inpatient stroke rehabilitation, we explored factors influencing implementation when moving from an explanatory trial under ideal conditions to a more pragmatic trial under real-world conditions. We identified implementation strategies and practical actions for implementation into routine inpatient stroke rehabilitation.

**Methods:**

Context and perspectives of delivering higher-intensity walking exercise in the Walk ‘n Watch (WnW) pragmatic trial were compared and contrasted with its predecessor, the Determining Optimal post-Stroke Exercise (DOSE) trial. The PRECIS-2 tool was used to quantify trials along the explanatory-to-pragmatic continuum. A sequential qualitative integrative approach compared perspectives from semi-structured interviews conducted with therapists and managers in the WnW trial (n = 18) to previously published therapist perspectives from the DOSE trial (n = 15). The Consolidated Framework for Implementation Research (CFIR) was used deductively. The CFIR-Expert Recommendations for Implementing Change (ERIC) matching tool was used retrospectively to identify key implementation strategies, operationalizing to practical actions employed during WnW protocol implementation.

**Results:**

PRECIS-2 analysis demonstrated a shift towards greater pragmatism (mean (SD) domain score 2.8 (1.4) vs 4.3 (0.7)). In both trials, therapists were motivated to deliver the protocol, despite differing belief systems and staffing challenges. In the WnW trial, therapists demonstrated greater readiness for change, actively implementing protocol principles whilst modifying delivery to meet needs. Managers and champions played an important role in supporting decision-making and systems-level compatibility. Ten ERIC strategies were identified focusing on evaluating needs, ensuring team readiness, promoting adaptability and ongoing engagement. Practical actions included optimizing innovation-context fit, fostering a collective commitment and focusing on communication.

**Conclusions:**

Capitalizing on a unique longitudinal opportunity, we report consistent, evolving and emerging factors that influenced implementation, highlighting the importance of iterative consideration of context and perspectives along the explanatory-to-pragmatic continuum. Our example indicates that higher-intensity walking exercise protocols hold promise for widespread adoption in stroke rehabilitation, with local modifications for optimizing innovation-context fit. Leveraging our relatively rare pragmatic trial, we identified implementation strategies and practical actions to provide tangible support for future implementation efforts. For long-term sustainability, economic factors require consideration.

**Trial registration:**

www.Clinicaltrials.gov ID: NCT01915368.

www.Clinicaltrials.gov ID: NCT04238260.

**Supplementary Information:**

The online version contains supplementary material available at 10.1186/s43058-025-00812-y.

Contributions to the literature

- Using a novel sequential qualitative integrative approach enabled valuable insights into the implementation of an innovation and its transition from explanatory clinical research to pragmatic application in usual clinical care.
- Consistent use of the Consolidated Framework for Implementation Research (CFIR) allowed comparative exploration of the static and dynamic contextual factors and perspectives influencing implementation over time.
- We go beyond identifying barriers and facilitators to implementation and take an interpretive step to translate the perspectives of therapists and managers in a pragmatic trial into implementation strategies and practical actions to facilitate future implementation efforts.

## Background

Clinical trials are commonly explanatory trials that test the efficacy of an innovation under ideal conditions [1–6]. The well-controlled environment of an explanatory trial maximizes internal validity so that group differences can be attributed to the innovation. However, stringent eligibility criteria may reduce the generalizability of the results. In addition, resources that are often available for operating the trial (e.g. extra staffing) may reduce the feasibility and sustainability of the protocol when implemented in usual care. In contrast, pragmatic trials, make up less than 5% of clinical trials [5, 6] and test the effectiveness of an innovation under usual care. Pragmatic trials must optimize an innovation for real-world conditions, which could include staffing challenges (e.g. high turn-over, varying experience, competing demands), as well as diverse patients with multi-morbidities. Pragmatic trials enable the results to be generalized to a broad spectrum of patients and can facilitate the implementation of the protocol in today’s clinical setting.

Current studies demonstrate that walking exercise intensity remains low in inpatient stroke rehabilitation practice [7, 8]. Previously, we completed a six-site explanatory randomized controlled trial (RCT) i.e. the Determining Optimal post-Stroke Exercise (DOSE) trial [9]. The DOSE trial investigated the efficacy of a complex structured, progressive protocol to deliver higher-intensity walking exercise in Canadian inpatient stroke rehabilitation units under ideal conditions. In this multi-site explanatory trial, one experienced front-line therapist (or a trained backfill) delivered the innovation per site, was mentored by the research team and had dedicated time for the protocol. The protocol demonstrated a significant and clinically meaningful improvement in walking capacity after the four-week inpatient innovation compared to usual care, and these gains were maintained at 12-months post-stroke.

Understanding the factors influencing implementation can assist in developing actionable strategies to facilitate adoption. Using qualitative methods, we studied the factors influencing the implementation of the higher-intensity walking exercise protocol in the DOSE trial, delivered under ideal conditions, using the Consolidated Framework for Implementation Research (CFIR) [10, 11]. However, it is useful to understand the factors influencing implementation and sustainability of the protocol as part of usual care. Recently, we undertook a pragmatic RCT i.e. the Walk ‘n Watch (WnW) trial which demonstrated the effectiveness of an adapted version of the higher-intensity walking protocol (WnW protocol) in usual care [12, 13]. Over 85 front-line therapists were involved in delivering the innovation across 12 sites [13]. Therapists delivered the protocol whether they were experienced or not, and no matter whether they attended the original one-time training or were trained by utilizing peers or other resources. Behaviour of health care professionals can be a substantial source of variance for effective and reliable uptake of an innovation [14]. Features of the healthcare setting can also impact implementation success [15, 16].

The aim of this study was to explore factors influencing implementation of this higher-intensity walking exercise protocol, comparing and contrasting contextual factors and therapist perspectives from an explanatory trial delivered under ideal conditions [9] to a pragmatic trial delivered as part of usual care [13] within inpatient stroke rehabilitation.

The objectives of this study were to:

1. Quantify the DOSE and WnW trials within the explanatory-to-pragmatic continuum.
2. Explore perspectives of therapists and managers in implementing the WnW protocol into usual care as part of the pragmatic WnW trial.
3. Compare perspectives from the pragmatic WnW trial to previously published perspectives [10, 11] acquired from implementing the protocol under ideal conditions as part of the explanatory DOSE trial [9].
4. Retrospectively identify strategies and operationalize to practical actions for real-world implementation by retrofitting to implementation activities deemed successful in the WnW trial.

## Methods

This paper compares and contrasts contextual factors and staff perspectives from the pragmatic WnW trial (Clinicaltrials.gov ID:NCT04238260) with its predecessor, the explanatory DOSE trial (ClinicalTrials.gov ID:NCT01915368).

### Context

The DOSE trial was a phase II multisite RCT conducted in Canada which investigated the efficacy of delivering a higher-intensity walking protocol in inpatient stroke rehabilitation between 2014 and 2018 [9].

To progress, from 2021, the WnW implementation phase III trial used a multisite stepped-wedge cluster RCT design to investigate the effect of introducing an adapted version of the protocol (i.e. WnW protocol) into usual care in several Canadian rehabilitation units, as described in Peters et al. (2023, 2025) [12, 13]. In the usual care stage, therapists continued to deliver usual care physical therapy on their inpatient stroke rehabilitation unit for a randomized period. In the implementation stage, the WnW protocol was implemented and replaced usual care for all eligible patients. The protocol focuses on completing a minimum of 30 min of weight-bearing, walking-related activities that progressively increase in intensity informed by activity trackers measuring heart rate and step number within physical therapy sessions.

The main contextual factors for both trial designs are summarised [based on full descriptions using PRECIS-2 domain headings presented in Additional file 1]. For the WnW trial, adaptations were made pre-implementation to the training, screening, and protocol used in DOSE in response to research findings and to optimize innovation-context fit for use in real-world conditions. The main similarities between the studies include protocol content (only minor changes in step targets and aligned to baseline Six Minute Walk Test (6MWT) for WnW) and trial outcome measures, follow-up and analysis. There were several differences. Major changes for the WnW trial included: more trial sites (12 vs 6) especially increasing the proportion in smaller cities including rural catchments; broadening of the eligibility criteria and removal of the graded exercise stress test; protocol training (one-time video-conference rather than in-person), screening and delivery was undertaken by *all* rehabilitation unit front-line physical therapists and rehabilitation assistants rather than being the responsibility of the research team and an experienced front-line therapist; rehabilitation unit managers were directly involved in trial organisation and adherence with some sites having a designated practice leader act as a ‘site coordinator’; and the rehabilitation unit was responsible for onboarding new staff to deliver the protocol. Minor differences included changes to training and technology hardware.

### Design

The PRECIS-2 tool was used to quantify the DOSE and WnW trials on the explanatory-to-pragmatic continuum [17].

Semi-structured interviews were used to explore perspectives of therapists and managers in implementing the WnW protocol into usual care in their stroke rehabilitation unit. A sequential qualitative integrative approach, where findings from two qualitative strands were combined for a unified interpretation (QUAL – QUAL) [18], was used to compare and contrast interview findings from the WnW study with those of the DOSE study. In both studies, the theoretical underpinning frameworks were the Normalization Process Theory (NPT) and CFIR. NPT can be used to understand how new innovations become integrated and sustained within usual care [19]. The CFIR is one of the most commonly used determinant frameworks and provides a menu of constructs, within domains, that have been related with effective implementation [15, 20]. Here, the refined compilation of implementation strategies from the CFIR-Expert Recommendations for Implementing Change (ERIC) project was applied retrospectively to identify key implementation strategies [21].

Manuscript preparation was guided the “Consolidated criteria for reporting qualitative research (COREQ)” [22] [see Additional file 2 for completed checklist].

### Data collection and analysis

#### PRECIS-2

Three researchers (SHH, SP, JJE) independently scored the nine PRECIS-2 domains using a 5-point Likert scale (1: Very explanatory – 5: Very pragmatic) for each trial considering the contextual factors outlined in additional file 1. Discrepancies were resolved by consensus. Domain scores were visually depicted on a PRECIS-2 wheel and summed, with a mean (SD) PRECIS-2 score calculated over the 9 domains. Higher scores indicate greater pragmatism and closer reflection of usual care.

#### DOSE therapist interviews

Published qualitative findings of therapists’ perceptions of delivering a higher-intensity walking protocol during the DOSE trial, derived from CFIR based framework analysis of semi-structured interviews, were collated from Connell et al. (2018) [10]. Factors influencing implementation were reallocated to best fit updated CFIR domains and constructs [20], as DOSE findings were coded according to the original CFIR [15].

#### Walk ‘n Watch (WnW) interviews

Therapists and rehabilitation assistants who had greater than two weeks of experience delivering the WnW protocol to more than one patient during the WnW trial, and managers of the involved rehabilitation units, were invited to participate in one semi-structured interview. Potential therapists and rehabilitation assistants were staff who participated in a previous survey study regarding the implementation of the WnW protocol [23], and unit managers, who agreed to be contacted for future related research**.** All trial sites were invited (10 English- and 2 French-speaking). All participants provided informed e-consent and received an honorarium to compensate for their time. Ethical approval was obtained by the relevant University boards.

Interviews aimed to capture the dynamics, challenges, and modifications of implementing the WnW protocol. The NPT [19] and CFIR [15, 20] were used in the development of the interview guide based on the guide used for the DOSE trial [10]. The interview guide was reviewed, revised and piloted by SSP and BO [see Additional file 3]. A French version of the interview guide was translated by a bilingual French native speaker (BO) and verified by a bilingual English native speaker (KLB). Interviews were conducted by SSP and BO over Zoom and lasted between 30 and 60 min. Participants were aware that the interviewer was not a direct part of the WnW trial management team, and that an honest perspective was wanted to learn lessons for implementation, with criticisms welcome. Interviews were recorded, anonymised and transcribed verbatim. The French interviews were translated to English, and verified (BO).

Interview transcripts were imported into NVivo 14 (Lumivero, USA). Framework analysis was undertaken using the updated CFIR to code data deductively, with additional free codes developed where needed. To establish a shared understanding and interpretation of the coding framework, all coders started by coding the same transcript. The coded transcript was compared and any variance in interpretation of data and application of codes was discussed to reach consensus. The remaining transcripts were coded by two coders independently, with SA and LC completing half each along with a second coder (SHH, LS, SJD, PT). The coding for translated French interviews was reviewed (BO). Main findings were collated according to the relevant updated CFIR domain and constructs, together with illustrative quotes, and agreed.

### Data integration and synthesis

Analysis from the WnW trial was contrasted and compared against the findings from the DOSE trial for the PRECIS-2 tool and interview findings. Using the CFIR framework, for the interviews, factors emerging from WnW analyses were considered alongside findings from the DOSE trial, with adaptations for innovation-context fit for real-world implementation noted. Situated within the relevant CFIR domains and constructs, factors common across studies were aligned (perspective may have remained consistent or evolved) and new factors were included. Integrated results were discussed and agreed.

The CFIR-ERIC matching tool (V0.53) was used in conjunction with the updated strategy matching file [24, 25] to match the relevant CFIR-based contextual constructs (updated or original when not directly mapped) to identify key implementation strategies. Transcripts were reviewed to identify evidence of application of the strategies and operationalized to practical actions for deliverers and managers implementing the WnW protocol by SA and LC, then discussed by the team and agreed.

## Researcher characteristics and reflexivity

Interviews were conducted by SSP (English) and BO (French). SSP is a research assistant and has a Masters in rehabilitation sciences, with three years of experience in conducting qualitative research in a variety of different healthcare settings. BO is an occupational therapist and doctoral student in rehabilitation science, with nine years of experience in conducting qualitative research in rehabilitation. Analysis and interpretation were led by two clinical-academics (SA and LC) from the UK, both experienced researchers and physical therapists in stroke rehabilitation, who were independent of the WnW trial management team. LC was involved with the DOSE qualitative research studies and had valuable insight into therapist and patients’ perspective of factors influencing implementation arising during this explanatory trial. Four further Canadian researchers with physical therapy (SHH, LS, SJD) and kinesiology (PT) backgrounds, who were part of the WnW primary research team, were also involved in analysis and interpretation of the data. Their role in WnW trial management allowed valuable contextual insight however they remained cognisant of potential bias.

## Results

### PRECIS-2

**Fig. 1:**
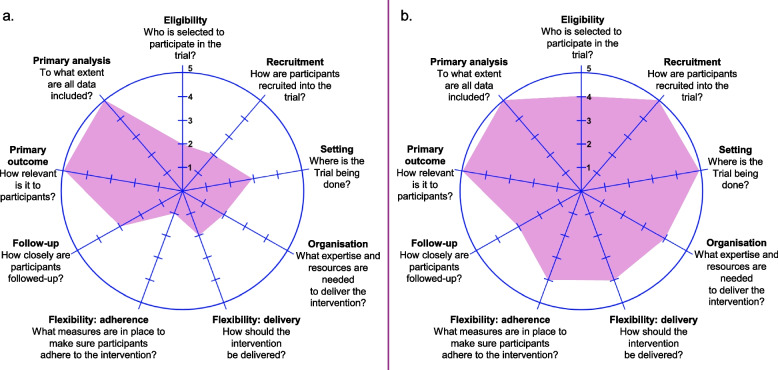
PRagmatic-Explanatory Continuum Indicator Summary–2 (PRECIS-2) wheel. Visual representation of summarised findings under nine domain headings for the a) DOSE trial [9] and b) Walk ‘n Watch (WnW) trial [13]. Scales range from 1: Very explanatory – 5: Very pragmatic. Higher scores indicate greater pragmatism and closer reflection of usual care. [Adapted from 'The PRECIS-2 tool: designing trials that are fit for purpose’, Loudon et al., Pg 3, 2015 [17], with permission from BMJ Publishing Group Limited]

Domain scores are visually represented in the Fig. 1 for the DOSE trial (a) and WnW (b). The DOSE trial had a summed score of 25/45 and mean (SD) PRECIS-2 domain score of 2.8 (1.4) and the WnW trial had a summed score of 39/45 and mean (SD) PRECIS-2 domain score of 4.3 (0.70). Findings demonstrate the shift towards greater pragmatism as the trials progressed across the explanatory-to-pragmatic continuum.

### Interviews

#### Participant characteristics

Thirty-two staff (28 of 47 who completed the WnW survey study, and 4 unit managers) were invited to participate in the current study in May 2023. These were individuals who agreed to be contacted for future related research, and represented all 12 sites. There was no overlap with WnW and DOSE participants. In total, fourteen therapists and four managers consented and were interviewed between June to December 2023. Their perceptions were compared and contrasted with those of fifteen therapists (including 3 rehabilitation assistants) from the DOSE trial, which was conducted between November 2016 to January 2017. In brief, WnW interviewees were all registered physical therapists and female. There was no rehabilitation assistant or male perspective garnered, although invited, unlike for the DOSE qualitative study. Interviewees in this WnW qualitative study were similar in age (50% between 30 – 39 yrs) to those in the DOSE qualitative study (mean age 37y), and a similar level of qualification (56% vs 50% Master’s level). Therapist and manager interviewees had slightly more years of experience as health professionals in WnW (Therapists: 16 ± 8, Managers: 20 ± 9) than those in DOSE (12 ± 10), as well as years of experience working with stroke patients (WnW Therapists: 10 ± 8, Managers: 13 ± 4 vs DOSE Therapists: 9 ± 8). In DOSE, some interviewees may have delivered the protocol twice daily (DOSE2 treatment arm).

#### Factors influencing implementation for therapists

Factors reported as influencing the delivery of higher-intensity walking exercise from WnW interview analysis were aligned with those from its predecessor DOSE trial, with new factors identified, and are presented in Tables 1, 2, 3, and 4 according to relevant updated CFIR domains and constructs. One free code ‘family’ was assigned under the outer setting domain, consistent with previous work [11]. Illustrative quotes from therapists (T) and managers (M) from the WnW trial are provided. A summary follows.

**Table 1:** CFIR Individual domain

Role	DOSE trial	Innovation-context fit adaptations for real-world implementation	Role	WnW trial	Illustrative quotes
*Therapists*	Most individuals were in the preparation or contemplation stage of change. Some recognised their practice had already changed. Others still felt they would “step back” to their everyday clinical practice. (M)	All unit therapists trained and encouraged to use the protocol, rather than 1 specified front-line therapist	*Therapists*	Most therapists felt motivated to use the protocol, felt their practice had changed, and will continue to use, many applying the principles of the protocol however modifying to suit their needs. (M, O)	*T08: “I think getting people on their feet for that 30 min duration is important and after having implemented the protocol and really seeing people improve at a fast rate, I would keep doing it for sure.”* *T05:” I would still implement that when we have stroke patients, even though we’re not technically doing the Walk ‘n Watch study, like I would still do that now with a majority of my stroke patients.”* *T15: “I know I try to use the concepts. I try to get that heart rate up. I’m not necessarily monitoring it …but I am trying to push for that intensity, … week to week, that sort of thing. But the concepts, yes.”*
			*Managers*	Managers recognised and facilitated the shift in practice over time for their units but realised this often involved modified use of the protocol. (O) Acknowledgement some therapists may revert back to their previous clinical practice. (M, O)	*M16: “We know it takes years and years of research to actually get part of like a clinician’s everyday … so it is trying to encourage staff, like let’s just keep at this, right. Just 30 min between us, between the rehab assistants, like let’s shoot for that 5 times a week, so yeah. So I think the buy in is ongoing, but I think it is getting better.”* *M16: “So they might not be doing all the pieces that we had to do with the study, but I think when talking to them, it’s changed their approach to therapy.”* *M03: “Sometimes what happens too, and again with any change we’re trying to make, is that the pull to old practice is really strong and just trying to change your ways is difficult.”*
*Therapists*	DOSE fits better with some people’s belief system than others due to conflict with quality of movement vs quantity of movement.(M) Some people’s beliefs changed once they had trialled the innovation. (M)	Similar content of protocol, minor change in walking steps targets	*Therapists*	WnW fits better with some people’s belief system than others creating areas of ‘tension’ (e.g. Scope of practice: simplicity of the protocol, quality vs quantity of movement, who was suitable for the protocol). (M)	*T04: “So my role as a physical therapist is kind of sidelined because of the volume that's required … It's irritating to say to yourself: "Well, that's it, I'll just have to be a walking assistant."* *T05: “I think it was just the wider thought process where it’s like, we don’t need to make things as complicated as sometimes we do, and it’s just a matter of actually getting steps in … it primarily was realizing that sometimes it’s okay to simplify things and just walk for half an hour.”* *T05: I struggled a little bit with trying to figure out quantity versus quality … So wrapping my head around trying to balance that, where it might not need to be perfect, but maybe it is more important to get steps and steps and steps in. And then by getting that more intensity, that can sometimes fix some of those problems that maybe we’re spending more time on, kind of nit-picking before actually getting into it.”* *T14: “It can include too low of a level of patient…when I think about the physicality that it took for us to actually apply the Walk ‘n Watch protocol to those patients … I’m worried that it would make people take on too much of a physical strain on themselves, just trying to meet that protocol.”*
			*Managers*	Managers were aware of ‘tensions,’ and in some rehab units time was required to find common ground to help shift practice. (M, O)	*M11: “There was a lot of concern at the beginning … it was actually like a huge uproar … we kind of found our common ground … we can just change the way we do things but not as drastically.”*
*Therapists*	Therapists gained confidence to “push people harder” due to: The graded exercise test, seeing patients able to work harder, and using heart rate monitors and step counters as objective measures.(C, O)	Screening undertaken by therapists using chart screen/6MWT/blood pressure. Graded exercise test removed for pragmatic reasons. Heart rate and step counters remained as real-time objective measures	*Therapists*	Chart review and clinical testing for screening deemed appropriate. (C) Therapists gained confidence to “push people harder” by seeing the patients safely complete the 6MWT, able to work harder, and using heart rate monitors and step counters as objective measures. A few therapists voiced concerns that missing targets could potentially demotivate. (C, O, M)	*T05: “Like the screen … I think it was relevant for sure … you always have to do your due diligence as a clinician to monitor that [blood pressure] as you’re going.”* *T10: “At first it was a little nerve wracking for me to … push someone beyond what they would probably be doing … before they had their stroke, but I was actually impressed. A lot of them could meet that kind of challenge … think it’s more having the confidence that even though all these folks are medically complex and a lot of them are elderly that they can reach these intensities and how much difference it makes in their recovery.”* *T01: “It has shown the immediate feedback that I’m looking for and it’s made me feel more confident.”* *T04: “Not reaching those targets is discouraging … my patient, who was more hemiplegic, was someone whose relationship with performance was very important, so it was discouraging for him.”*
			*Managers*	Chart review and clinical testing for screening deemed appropriate. (C) Some managers felt patients haven’t been pushed hard enough previously and that the protocol provided a good opportunity to demonstrate that patients can meet the challenge and saw the value of using real-time objective measures to aid this.(C, O)	*M16: “Like most people were medically well enough, we had to sometimes clear it with the physician.”* *M06: “I truly feel like we don’t typically push our patients as hard as they can be pushed…I think it’s been really good to show our therapists that patients can tolerate it more than, perhaps, we expected.”* *M11: “I think the Walk ‘n Watch study has actually changed our focus to looking more at their heart rate and working towards specific targets, so it allows the therapist that little bit of room to push a patient even beyond when they think they’re tired.”*
*Therapists*	Leadership engagement recognised as important to support resources required. (O)	Engaged with practice leaders and supervisors for trial approval and to organize protocol training, rather than just for trial approval	*Managers*	Leadership and colleague buy-in recognised as important. (O) Consideration was given by a manager regarding the economic considerations required for leaders/decision-makers. (O)	*M03: “The financial piece as well … because that’s always a question from finance, how much is this going to cost us? The fact it wasn’t going to be something removed or something added, it was just going to be: we’re not spending any more time with this patient’s time slot, we’re just changing what we are doing, so don’t worry that we’re adding more staff hours to the process … we’re not keeping patients any longer to participate in the study, … so questions that leadership had … were able to be clearly answered and reported back and there was good support from leadership here.”*
		Some sites had a designated practice leader act as a ‘site coordinator’	*Therapists*	Therapists found having a site coordinator valuable.(O)	*T07: “The ingredient, I think, was … to have a coordinator available on site.”*
			*Managers*	Managers found having a site coordinator valuable.(O)	*M03: “Our coordinators have also been quite keen, again, kind of reminding staff about checking in on a daily basis about new stroke admissions and who’s appropriate.”*

Characteristics of the individuals based on the COM-B system are indicated: Capability (C), Opportunity (O), Motivation (M) [20, 25]

**Table 2:** CFIR Innovation domain

Construct	Role	DOSE trial	Innovation-context fit adaptations for real-world implementation	Role	WnW trial	Illustrative quotes
**Evidence-base**	*Therapists*	Practical experience of using the innovation tended to outweigh publications Some mention of the importance of having underpinning research	Recommendations on intensity from credible organizations (e.g. Academy of Neurologic Physical Therapy) were presented	*Therapists*	Many referred to the underpinning research and best practice guidelines Practical experience facilitated translating theory to practice	*T15: “I know the research that’s out there definitely supports a higher intensity and it has for a long time. I think the more research that’s being done is continuing to say, intensity, intensity.”* *T01: “As a therapist, I know that the best practice has us looking at dosage and making sure we’re hitting therapeutic targets. And with a program like the Walk ‘n Watch that helps to give you a program to follow and targets to hit, it seems to have made it a little bit easier to translate that knowledge of what best practice is into actual clinical applications for the day-to-day setting.”*
				*Managers*	Some highlighted the impact of research in driving shifts in practice	*M03: “The more research we have to validate certain approaches, the more people will, I think, change their approaches to practice.”*
**Relative advantage**			Strong focus on the effect of higher intensity on walking recovery	*Therapists*	Some recognition of quicker gains, cardiovascular benefits, implications on other domains (motor learning) and patient behaviour long-term. However, not recognised by all	*T08: “You can see it when people are making these gains, making these improvements at a pretty quick pace. Like it’s impressive to see people really reach their targets like faster and faster.”* *T02: “It's true that walking helps… It's good for everything else, for energy, for all vital functions … And for a patient who's had a stroke, … who's got problems on all sorts of levels, it's definitely good.”* *T04: “By working on the cardiovascular system, I imagine that in terms of motor learning, in terms of the brain, having this oxygenation … can create better encoding, in some ways.”* *T09: “Unfortunately we had very few … that qualified. A lot of them were … what we call our walking wounded, cognitively-impaired and walking, so they had no gait deficits.”*
				*Managers*	Some recognition that wider benefits are often overlooked and an ambition by some to provide education across rehabilitation pathway	*M06: “It’s challenging on an inpatient rehab unit because there is so much to work on and with the ultimate goal of helping the patient get home sooner than later … sometimes the aerobic component is overlooked.”* *M16: “One of the things that is on my to-do list is to use that training to do some more with our outpatient department, … I think that they should be also educated because they’re now seeing stroke patients, after they’ve left rehab, and so to continue with the concept of high intensity gait training.”*
**Adaptability**	*Therapists*	Protocol needs to be adaptable for clinical reality (e.g., more focus on upper limb/education for some patients)	Training included discussion of strategies to make time for competing therapy needs (e.g. use of rehabilitation assistants, group programs)	*Therapists*	Protocol needs to be adaptable for clinical reality (e.g. more focus on upper limb/education for some patients) as competing therapy needs made prioritising treatment sessions difficult, especially with time constraints Some therapists noted use of strategies to modify for clinical reality	*T09: “It’s very challenging to meet all of their other inpatient rehab needs, and just achieve those steps, because you don’t have time.”* *T18: “So, carrying items, pushing shopping carts, pushing weighted carts, pushing things, dragging things, picking stuff off the ground, like walking and lifting things and moving things around, yeah that kind of stuff for the higher level patients you could do a little bit more with.”* *T01: “We’d look at totalling the amount of time within the day for people, so it wasn’t just necessarily within the session. If we were able to get 15 min one session …, my next session with them I’d consider going back to the protocol and seeing if I could get another 15 or 20 min in there.”*
				*Managers*	Protocol needs to be adaptable for clinical reality (e.g., more focus on upper limb/education for some patients) as competing therapy needs made prioritising treatment sessions difficult, especially with time constraints	*M11: “The other concern was just our need to get the other things done. So training the family on some safety things, like getting in and out of a car, and how are you going to help them dressing, and how to get up from a fall, and all those other things that we need to fit into our therapy.”*
	*Therapists*	Therapists thought “pre-gait” activities were essential, though recognised doing this first may reduce intensity	Recommendations from credible organizations (e.g. Academy of Neurologic Physical Therapy) that pre-gait activities were not needed were presented	*Therapists*	Some therapists were concerned that some activities (“pre-gait” or “advanced walking”) were essential but resulted in fewer steps	*T04: “… because he can walk with a cane and then take millions of steps, but in the end, there's almost no weight on his hemiplegic side, so it's a motor learning process, you have to walk at a slower speed to do it, so you take fewer steps.”* *T02: “The easiest way to reach it is to walk in a really straight line, and then not to stop, but personally, I think it's really important to walk in all kinds of ways: backwards, sideways, cross-stepping, in a line, on the stairs, like, all kinds of walking.”*
				*Managers*	Some managers noted using rehabilitation assistants for “pre-gait” activities or delivery of the protocol	*M11: “To prep the body for exercise that we could do before we actually start the treatment program, so we would delegate some of that to our therapy assistants … so that when we went in to actually do our therapy session, it was all focused on, we were ready to start the walking.”* *M16: “It was really great having our rehab assistants trained because they could then take on a session or two a week and then the therapist could do some other things with them, so then they could be working on transfers or upper extremity, or working on that little bit of quality with gait that they wanted to.”*
			Encouragement to use the protocol with eligible patients, even if the patient did not consent for the outcome measures	*Therapists*	Protocol applied with patients with other health conditions or in other settings, usually in modified versions	*T14: “We definitely, we apply it to everyone. Spinal cords, MS, yeah.”* *T02: “I did it for outpatients. They weren't included in the study … but, of course, we don't do the complete, complete, complete, complete protocol.”*
**Complexity**			Similar content in protocol, with heart rate target remaining the same and minor change in walking steps targets	*Therapists*	Several therapists used the principles for intensity rather than specific targets	*T15: “I can speak for myself, but I know I try to use the concepts. I try to get that heart rate up. I’m not necessarily monitoring it the way that I probably should, … but I am trying to push for that intensity.”*
				*Managers*	Principles for intensity used rather than specific targets	*M11: “I think it was more about educating the team that … 1500 steps might not be … you might not be able to get that because this patient is 96. But maybe you can still increase their steps from what they were doing … it wasn’t so much a push to get the steps outlined in the study but more a push to improve.”*
**Design**	*Therapists*	Therapists liked the structure and detail of the manual and paperwork, particularly tips and ideas Structured format helped support different therapists treating the same patients	Website was created for therapists to access training videos, examples for protocol, brief instruction sheets	*Therapists*	Therapists liked the structure and detail of the manual and paperwork, particularly tips and ideas Structured format helped support different therapists treating the same patients	*T09: “I liked having the manual, especially the example protocols of how to achieve those intensities … I really liked the supplemental information about how to assist, like gait phases, and how to increase intensity with like … the weighted vest, or stairs, … just all of those ways to meet the protocol.”* *T05: “I think we had enough of the basic information to figure out how it works. And the little quick … like cheat sheet cards …, that is super helpful when people are coming in to cover your caseload and they may not be super familiar with the protocol but it was easy for them to pick it up.”*
				*Managers*	Identified system-level supports that may facilitate e.g. prompts on electronic systems	*M03: “Electronic documentation, there could be mandatory fields as well, which … prompts behavior change … or practice change, and makes it easy to document the progress and see the progress as well.”*

**Table 3:** CFIR Inner setting domain

Construct	Role	DOSE trial	Innovation-context fit adaptations for real-world implementation	Role	WnW trial	Illustrative quotes
**Structural characteristics**	*Therapists*	Concerns regarding staffing to enable the (frequency and) duration of therapy outside of the study Shift required in how therapists prioritize treatment and buy-in from all therapists and managers when scheduling to allow for longer sessions	All unit therapists encouraged to apply the protocol with all eligible patients, rather than 1 specified front-line therapist allocated to deliver the protocol with just trial participants	*Therapists*	Staffing ratios and structures to enable the frequency and duration of therapy and complete documentation was a barrier for many There was difficulty balancing with other workload priorities (e.g. discharge planning, meetings) These factors could impact delivery and continuation of the protocol	*T13: “If you consider how the hospitals are right now, post-pandemic with healthcare provider burnout and like staffing shortages, adding an extra thing for therapists to do … once I started using it, I realized that it wasn’t so much extra to do.”* *T01: “I think the hardest bit for myself was the way we structure things with our rehab unit here. We don’t always have patients, say for, an hour total of therapy … trying to get a 30 min chunk of time, or hitting the 30 min thresholds … with meetings, family conferences, home visits, that sort of thing, that was a little bit difficult to implement on a daily basis, but we did our best.”* *T08: “Since stopping implementing the protocol in terms of the research study, we’re still using it, but I’d say not as often. And part of that is maybe because our workload has increased over just because our hospital is so over capacity … but I think once our staffing, or … our caseload levels get back to normal, we will see an increase in how often we’re using it.”*
				*Managers*	Managers recognised staffing ratios and structures to enable the frequency and duration of therapy were a barrier but often not compatible with capacity Recognised there was difficulty balancing with other workload priorities (e.g. education, discharge planning, meetings)	*M11: “Staffing is a nightmare. Our staffing has been very difficult in the past 6 months especially, we’ve had a lot of turnovers, so there’s not capacity to add extra sessions or extra treatment.”* *M06: “So working inpatient rehab … we have no control over when they show up to the gym … So, you might have a patient booked for half an hour, you might get 20 min. Because they showed up late … so, I think in the beginning, staff may have stressed a little bit about “I should hit this target, I’m supposed to do it in 30 min, but we only had 20, so we didn’t hit the target, am I in trouble?”* *M16: “I was a little bit worried about buy in and having clinicians commit 30 min of time to just walking, because I know, and for myself as well, that there’s other things that we need to work on.”*
			Therapists encouraged patients to complete ‘out of therapy session practice’ (with guidelines for safety), which was not specified in DOSE	*Therapists*	Some therapists empowered patients to do out of therapy session practice	*T05: “What we do during a therapy session is essentially a drop in the bucket as far as what they’re doing in the other 24 h in the day…it’s trying to empower people outside of that therapy time…if outside of this therapy session, like here’s your watch, you got this, you could get this in either with family assisting, people had something more objective to shoot for…Or like, on weekends, they would come back and say like oh I got this many steps, and they would never have done that before.”*
				*Managers*	In one unit, a manager reported a shift in practice by the wider team to encourage out of therapy session practice, facilitated by training	*M11: “We have the occupational therapists, we even trained them, even though they weren’t delivering the protocol but we trained them so that they might be a consistent on the weekend to actually help with technology so that when there was the therapists that weren’t trained, we could still continue.”* *M11: “I think even seeing our nursing team, … seeing them say “well, look at your watch, you haven’t even gotten your steps today, let’s walk to dinner, let’s walk here” I’m noticing those little changes show me it’s working. It’s just kind of become part of normal treatment for some.”*
**Communications**	*Therapists*	Communication important to ensure treatment schedules work to allow for longer sessions	Therapists engaged with practice leaders and supervisors, and some sites had designated practice leader act as a ‘site coordinator’ and as primary contact for therapists, rather than direct contact with the research team	*Therapists*	Regular communication important to share successes and problem-solve	*T07: “It was fun to talk about what was going well, our difficulties and all that, and it also enabled us to adjust our interventions, while optimizing our care for the user.”* *T09: “I think the weekly huddles, the reminder that this is what we’re doing, and this is why, and providing that handout on best practice and how to incorporate it into the training session.”*
				*Managers*	Regular communication important to share successes and problem-solve	*M16: “Regular communication was obviously a huge thing for us and we, as a group, huddled once a week regardless of the study, so we had our own thing, but that would be a time for us to talk about the study, to talk about any issues, to talk about eligibility, who might need to be screened, and things like that.”*
**Culture**	*Therapists*	Recognition that this type of innovation will not be suitable for all (especially elderly with comorbidities)	Screening by therapists, rather than by the research team	*Therapists*	Decisions on starting the protocol were sometimes made by therapists based on patient demographics and characteristics	*T12:* “*Potentially I was being a little bit more selective. I was choosing patients that definitely wanted a goal of walking … but also had pretty good strength … it was more coordination deficit, low endurance. Those were sort of, my target groups. Especially people who were living on their own and wanted to go back to living on their own.”*
				*Managers*	Aware decisions on starting and using the protocol were also sometimes made based on therapists’ capability and they had a role in supporting decision-making	*M11: “I was finding that there was certain staff who would [say], “oh no no no, they’re not ready, they’re not ready”* *M16: “There’s also just like inherent differences in therapists’ strength, right, and physical ability to work with patients as well as their comfort and their experience”* *M16:* “S*o sometimes I would work with that physio with the patient and try to show some strategies that might allow that patient to be a one assist, or is there a difference in handling and things like that*”
			All unit therapists trained and encouraged to use the protocol, rather than 1 specified front-line therapist	*Therapists*	Value seen in working together as a group	*T10: “This is like something we’re doing as a group, so then it was a little less nerve wracking for me because it was something that we’re all doing as a group in our workplace.”*
				*Managers*	Value seen in working together as a group	*M06: “We met as a group…to discuss it and whether or not we thought that as a team, we had the capacity to do it…we just met as a team and thought it was something important enough to participate in as a team and went for it.”*
**Available resources**	*Therapists*	Need for graded exercise test, and ideally equipment (Heart rate monitors, step counters, treadmills, harnesses)	Heart rate monitors (Garmin watch) and step counters (Fitbit Inspire) provided to units for use with all eligible patients, with watch given to patients to keep if consented for outcome measures. All therapists to use technology, rather than specified front-line therapist Not all units had specialised equipment (e.g. harnesses)	*Therapists*	Although some liked the idea of technology there were some reservations, and practice was required for many to gain confidence in use of heart rate monitors and step counters Inaccuracies in measures caused frustration	*T01: “I liked the idea of using the tech as a simple and easy way to track peoples’ heart rates, their step count, and to be able to measure that target zone…”* *T13: “I felt until I actually starting doing it and saw that it wasn’t so demanding, the initial like, to start doing it with the first person, felt a little bit dreadful because I was just thinking, this is one more thing, …once we figured it out, it was good.”* *T14: “The technology when it works is brilliant, but, you know, if you can’t rely on it and you’re trying to hit a target, you need some other objective measures. So yeah, the technology wasn’t reliable enough.”*
				*Managers*	Recognised challenges with equipment often related to confidence and reported strategies used to help mitigate including rehabilitation assistant support	*M03: “I think some of the challenges was just getting the technology, like the watch and things like that and the step counter, things that people just weren’t as comfortable with. You can figure things out, but if you’re not comfortable or not used to the technology, it feels like it’s another step.”* *M16: “Sometimes the watch wouldn’t quite work, the Fitbit we had a lot of problems with, just because that super slow stepping…some therapists wore it on their leg and they tried to match the steps because then they could take a quicker step so that the reader would count it, so there was some trial and error in trying to figure out how do we actually count those steps…So we played around with things like putting it in the pocket, putting it on the ankle, on the shoe”* *M16: “Rehab assistants were really really good at like setting it [equipment] up for staff and they made a little cheat sheet that actually went around the patient’s neck, like a laminated card, just with a reminder of what buttons to press, and like it also acted as a reminder.”*
**Access to knowledge and information**			Training via video-conference with hands-on practice with heart rate and step counter, rather than in-person. Training recorded for future use. Website with resources (e.g. videos, quick instruction sheets) provided	*Therapists*	Training was seen as beneficial, including the recorded versions for self-managed online training	*T12: “Open discussion examples, we all got to practice. Yeah, thinking back, I felt like we had a good amount of time and practice to be able to then consider inputting it.”* *T08: “I essentially was given kind of like the protocol and information *via* handout and they had recorded a YouTube video of kind of the initial training…And so I watched that, but it was kind of not like official training, it was more like self-based studying… Like the coordinator at our site was there for the first couple sessions I did and like always there for questions. I didn’t get any like, again, like formal follow up training but I definitely was given a lot of assistance, if needed, when implementing the protocol.”* *T01: “I felt very comfortable with just the online and reading materials, but then having that person [colleague] there to help support as well was definitely beneficial… by the point that I came into it, our assistants were quite comfortable with it, …they were able to help me out the first couple of times we would use it as well… so, it was available to us informally on an as-needed basis.”*
				*Managers*	Managers found the recorded versions of the training useful, including for managing staff turn-over	*M11: “Adding the weights, the weighted vest, the weighted belts, our team really bought into that because that’s actually helping loading and its increasing heart rate, so definitely appreciated that.”* *M16: “The training was really great, [research team member] sent me the taped ones so that as we had new staff … just to train them.”*

**Table 4:** CFIR Outer setting domain

Construct	Role	DOSE trial	Intervention-context fit adaptations for real-world implementation	Role	WnW trial	Illustrative quotes
**Family involvement**			Therapists encouraged patients to complete ‘out of therapy session practice’ (with guidelines for safety) including family assistance if appropriate	*Therapists*	Family involvement deemed beneficial	*T18: “People that have family that are able to be here and help out and facilitate that happening, those ones do better.”* *T09: “There was one that for sure stands out, who probably pushed to walk and her family then pushed her to walk more…I think it’s those ones who had the family support with the information, you know, that the steps matter, that actually improved a lot.”*
				*Managers*	Family involvement deemed beneficial	*M16: “Like one of the patients bought all his family Garmin watches so they could try to increase their steps per day as well, and he sent them challenges and stuff, so he very much bought into it.”*

In both the DOSE and WnW trials, therapists generally expressed positive opinions about the innovation. They were motivated to deliver the protocol and gained confidence over time. Key facilitators remained including practical experience, protocol training (including its online format), associated materials, leadership buy-in and effective communication. However, differing belief systems resulted in persistent tensions. Continued barriers included staffing challenges such as competing therapy and workload demands, and staffing capacity. Managers and champions in the WnW trial played a pivotal role in mitigating many of these challenges by supporting decision-making and systems-level compatibility, though workforce shortages remained a significant barrier.

Since the explanatory DOSE trial, therapists’ readiness for change evolved, shifting from contemplating new practices to actively adopting them. Awareness of supporting evidence grew, and many therapists appreciated opportunities for support to bridge theory and practice. As predicted by DOSE therapists, modifications to the protocol use were made to align with individual and service needs. Technology challenges shifted from considering the need for resource to building confidence in its use. Nonetheless, frustrations with technology accuracy to measure walking exercise intensity persisted.

Newly emerging factors included recognizing the value of site coordinators as champions and working together as a group for implementation. A greater emphasis was placed on empowering patients’ out-of-session practice, supported by the wider care team and families. Therapists expressed varied beliefs about the simplicity of the protocol: while some appreciated its straightforward nature, others felt it constrained use of their clinical skills.

### Strategies and actions for implementation of the Walk ‘n Watch (WnW) protocol under real-world conditions

Ten key ERIC strategies identified using the CFIR-ERIC matching tool, with practical actions for deliverers and managers implementing the WnW protocol in inpatient stroke rehabilitation settings, are summarised in Table 5. These strategies focus on evaluating needs, ensuring team readiness for implementation, promoting adaptability and fostering ongoing engagement. Strategies were operationalized to practical actions by retrofitting to implementation activities perceived successful by therapists and managers in the WnW trial. These include optimizing innovation-context fit, cultivating collective commitment to implementation, and emphasizing robust communication and feedback mechanisms.

**Table 5:** WnW protocol implementation strategies gained retrospectively and practical actions for real-world implementation

ERIC strategy	Deliverers actions	Manager actions
1. Conduct local needs assessment	• Share insights (and data) related to the need for the innovation	• Collect and interpret data related to the need for the innovation
2. Assess for readiness and identify barriers and facilitators	• Share initial insights about the innovation • Review how the protocol fits with usual care • Identify and discuss barriers (including potential tensions) and facilitators to successful implementation and sustainment • Consider space and resources that might improve delivery including equipment, multidisciplinary team and family involvement etc	• Review how the protocol fits with usual care, along with service and organisational practice and priorities • Identify and discuss barriers (including potential tensions) and facilitators to successful implementation and sustainment • Consider space and available resources • Determine who needs to be involved or kept informed e.g. innovation deliverers, wider multidisciplinary team, patient, family, outpatients, decision-makers
3. Identify and prepare champions	• Clear person identified with appropriate experience, self-efficacy and willingness to perform champion role	• Identify and designate a champion • Allocate time/resources and delegate authority to the role and recognise achievements
4. Conduct educational meetings	• Actively participate in training and draw on expertise from colleagues • Take opportunities to practice and trouble-shoot use of innovation including equipment	• Engage the right people and promote enthusiasm about the innovation • Share evidence strength and relative advantage • Promote any wider benefits of innovation e.g. immediate and longer-term • Make use of online resources
5. Develop and distribute educational materials	• Develop local processes for screening patients effectively (using evidenced criteria and clinical judgment) and supporting delivery by a range of staff (including weekend and casual staff) • Make manuals, training resources etc. easily accessible	• Provide leadership and support for patient screening, training opportunity, and model best practices • Facilitate system-level solutions e.g. updating electronic documentation systems to reflect innovation use
6. Conduct local consensus discussions	• Recognise the value of working together as a group and outreach to other relevant groups	• Recognise the value of working together as a group and facilitate outreach to other relevant groups
7. Organize clinician implementation team meetings	• Actively participate in team discussions to refine the innovation and collectively address tensions and priorities • Reflect on conscious/unconscious bias that may influence practice	• Hold regular team meetings/workshops for implementation co-design and ongoing discussion on how staff are managing balancing tensions and priorities and to identify potential adjustments in practice • Promote teamwide engagement in the innovation to create a sense of shared responsibility and collective ownership
8. Promote adaptability	• Recognise modifications to delivery and consider their impact - Are the principles of the innovation retained? Consider a fidelity check • Adjust practice and schedules to suit innovation deliverer and recipient, and partner with colleagues e.g. assistants, supervisor	• Support deliverers in modifying the innovation to their specific practice environments while maintaining its principles • Consider a fidelity check at a service level • Highlight success stories to build buy-in and offer additional training or mentorship to staff who are resistant to the change • Facilitate practice and schedule adjustments to support staff capacity, capability and well-being • Facilitate strategies, such as using assistants and consider resources that might facilitate delivery
9. Capture and share local knowledge	• Build confidence in a new way of working and reflect on experiential learning • Embrace objective measures, making goals clearer and more attainable • Maintain engagement and motivation by sharing knowledge on varying activities, using technology, and find creative ways to keep both yourself and the patient motivated • Empower patients to engage in self-management where possible	• Boost staff confidence through active support • Encourage and facilitate the effective use and interpretation of objective measures • Foster a cultural shift (deliverers/wider team/relevant settings) to engage and sustain the way of working • Empower staff to support colleagues • Undertake periodic reviews and discussions to ensure staff remain committed to the innovation and provide recognition for those who consistently apply it
10. Obtain and use patients/consumers and family feedback	• Involve family when able and appropriate and seek feedback from patients and their family.	• Develop processes to collect and interpret feedback from patients and their families

## Discussion

We explored factors influencing implementation of a higher-intensity rehabilitation innovation, providing a unique longitudinal view by comparing and contrasting contexts and perspectives of an explanatory trial with a pragmatic trial conducted in inpatient stroke rehabilitation settings. Using the pragmatic WnW trial we expanded the focus by incorporating management perspectives alongside therapists’, adding a valuable systems-level dimension. As anticipated, PRECIS-2 analysis confirmed the deliberate intent toward greater pragmatism. While some factors influencing implementation remained consistent with a shift across the continuum, others evolved or emerged. Although the innovation underwent minor adaptation between trials, therapists and managers in the WnW trial further modified protocol use to address local contextual constraints, while striving to uphold the principles. Using the CFIR-ERIC matching tool, insights from therapists and managers identified 10 key implementation strategies employed during the WnW trial. These were operationalized into practical actions by retrofitting to implementation activities perceived to be successful.

### A shift towards greater pragmatism: factors influencing implementation

Enthusiasm for the protocol persisted even with adaptations for real-world relevance, for instance by involving all therapists in delivery and moving responsibility for screening, training and adherence to rehabilitation units. Workforce pressures unsurprisingly were a recurring concern in both trials. Globally, barriers such as limited staffing and time constraints, are well-documented obstacles to knowledge translation [26–28]. Encouragingly, teams found mechanisms to integrate innovation requirements into their clinical practice, amidst these realities. Strategic workforce planning, effective resource allocation and investment in staff training are consistently emphasized as solutions to implementation success, alongside investment in individuals and organizational culture [27]. These aligned with several implementation strategies and actions employed by managers during the WnW trial. Tensions persisted and emerged as therapists and managers grappled with differing beliefs regarding scope of practice (i.e. which patients are suitable, who should be delivering and how to balance priorities). Implementing standardized protocols while recognizing professional autonomy requires thoughtful consideration and must be context-dependent, given that health systems across countries adhere to varying professional standards and practices.

Therapists’ mindsets had evolved between the trials. In the DOSE trial, therapists were predominantly in a contemplation stage, evaluating the innovation’s pros and cons, gathering information, and deliberating adoption [10]. In contrast, the WnW trial saw entire rehabilitation units undergo a ‘shift in practice’ with therapists and managers advancing to action and maintenance stages. This progression is of note given higher-intensity therapy has been recommended in guidelines for some time, yet adoption has been slow [7, 8, 29, 30]. The sequential integrative approach illustrated this important shift in readiness to change. Enhanced staff awareness of the evidence-base, a collective commitment to meeting guidelines and the perceived value of the innovation likely contributed to progress, as found by others [15, 26, 27, 31]. This evolution may reflect dissemination efforts by the field as well as implementation strategies used in the trial. The findings emphasize the value of formal and experiential learning opportunities, and underscore how research engagement can improve processes of care and healthcare outcomes [32–34].

Although modified use of the protocol was common (see Tables 1, 2, and 3 for examples), therapists aimed to uphold its core principles for intensifying rehabilitation, and positive outcomes were observed [13]. Before implementation, to enhance feasibility step targets were adapted (in some cases reduced) compared to the DOSE trial [35]. Modifications enacted by therapists during WnW protocol delivery included a ‘push to improve rather than getting the steps outlined’. Modifications supported implementation, reflecting the dynamic nature of healthcare innovations that often require adjustment to fit local contexts and support uptake [36]. Single-session fidelity testing showed a substantial increase in step count in the WnW (1513 steps) compared to the usual care group (980 steps), with participants achieving, on average, 62% and 42% of their step targets respectively [13]. Similarly, the Quality in Acute Stroke Care (QASC) study, a nurse-led intervention targeting fever, hyperglycaemia and swallowing, demonstrated that implementing a structured care bundle influenced clinician behaviour change and improved patient outcomes, despite variable adherence [37]. While a dose–response relationship in rehabilitation is established [38], the threshold for optimal benefit remains unclear. Together these findings reinforce that strict fidelity may not always be necessary. A longstanding debate in implementation science concerns balancing fidelity with adaptation to local contexts [36, 39, 40]: excessive fidelity can impede uptake, whereas unplanned adaptations risk compromising essential elements. Our interviews highlighted practical challenges and tensions in applying the protocol in real-world setting. Although the protocol’s principles were communicated, greater clarity on core components versus adaptable periphery will be beneficial. [15, 36, 40]. A forthcoming paper will detail the core components of the WnW protocol.

### Strategies and actions for implementation in the real-world setting

A strength of this study is that it goes beyond identifying barriers and facilitators to implementation. It takes an interpretive step by translating the perspectives of therapists and managers into strategies and then outlines practical actions on *how* staff might contribute to implementation efforts, addressing a recognised need [41]. The ERIC compilation has faced criticism including its vague operationalization, with recommendations to detail the actors (i.e. who is enacting the strategy), actions (i.e. specific activities to support implementation) and relevance to specific phases [42–44]. Further, although active, targeted implementation strategies have demonstrated some effectiveness in bridging evidence-practice gaps, no single implementation strategy works universally across all circumstances or healthcare settings [45, 46]. Here we address these concerns by contributing a suite of strategies with practical actions for deliverers and managers to facilitate pre-implementation through to sustainment of the WnW protocol. Importantly, these were operationalized from actual implementation activities described by therapists and managers in a relatively rare pragmatic trial. For actions, we intentionally use terminology that may allow broader application when implementing other rehabilitation innovations, recognising that further research will be required to validate. Actions focused on optimizing innovation-context fit, cultivating collective commitment to implementation, and emphasizing robust communication will now be discussed.

We have detailed actions that target contextual considerations and collaboratively plan how the innovation may best fit with usual care. Understanding context is integral for successful implementation [16, 40]. Notably, a focus on aligning the innovation with existing service and organizational practices and priorities from the pre-implementation phase is proposed. Early attention on system-level factors such as strategic alignment, wide stakeholder engagement (including decision-makers), and resource availability, is vital for creating opportunities for both implementation and sustainment [47, 48].

Cultivating roles and relationships also plays a critical role in securing commitment for implementation and scale up [31]. Actions include engaging appropriate leaders and designating specific roles, such as a champion. Engaging local opinion leaders has been shown to be a prominent strategy [45, 49]. A model for optimizing champion contributions, offering guidance on identifying, preparing and evaluating champions, may serve as a valuable resource [49]. Working as a collective group and creating communities of practice, are identified as valuable and may help foster a culture of shared ownership which can enhance implementation and sustainment [27, 50]. Robust, multi-layered communication is another key element in facilitating implementation efforts [31]. The availability of resources, such as online access to manual use or standardized training, can support team readiness for implementation and strengthen fidelity [51, 52]. In the WnW trial, provided materials supported staff during implementation and onboarding (https://neurorehab.med.ubc.ca/walk-n-watch/). Ensuring these materials are widely accessible is necessary to sustain and enhance future implementation efforts. Effective communications should extend beyond the immediate teams, casting a wide lens on the potential impacts of the innovation. Professional networks, crucial facilitators of knowledge transfer [51], should be recognised and prioritized.

Going forward, some key uncertainties remain in enabling widespread implementation and sustainment. While many therapists and managers have expressed a desire to sustain delivery of the WnW protocol in modified versions, doing so at a systems-level likely requires establishing a clear value proposition for decision-makers. Evaluation summaries will contribute; however, an understanding of the financial implications is likely required, as highlighted by one manager in the WnW trial. A health economic analysis could be a crucial element for many health systems, providing insights into the comparative resource requirements and outcomes of the innovations for the individuals and organisations affected [53]. Another challenge involves how new developments in technology will be integrated to support protocol use. Both trials encountered technological inaccuracies in measuring walking exercise intensity, which lead to frustrations and influenced fidelity. While technology is advancing rapidly, identifying and integrating the most appropriate technology hardware within available resources is required.

### Limitations

The study involves two trials that started seven years apart. Using PRECIS-2 headings we described contextual factors relating to each trial; however, this approach offers limited insight into the external influences on innovation implementation (i.e. CFIR outer setting domain). As expected, political, economic and strategic landscapes underwent substantial changes over this period. A notable difference is that one trial occurred pre-COVID-19 and the other in the COVID-19 era. Given that sustainability considerations place greater emphasis on factors within the outer setting, future study design may benefit from including a description of factors impacting sustainment. Despite this limitation, the study highlighted important factors influencing implementation through the individual, innovation and inner setting domains. The unique longitudinal view taken also enabled identification of factors influencing implementation at different stages of the research continuum.

The use of the CFIR for analysis for both qualitative studies of the protocol enabled us to compare and contrast perspectives. However, the CFIR underwent an update during the interval between studies, with domains and constructs revised, removed or added [15, 20]. To enable sequential integration and ensure methodological relevance, we reallocated DOSE qualitative study findings under the updated CFIR domains and constructs. While this process required a degree of subjective decision-making, we were guided by CFIR publications to achieve best-fit [20, 25]. Further challenges were the limited compatibility of the CFIR-ERIC matching tool with the updated CFIR, as well as considering ongoing debates about its utility [42–44]. To address these, we leveraged our knowledge of CFIR constructs when carefully applying the tool and discussed and reached consensus on the relevance of the highest-ranking strategies. As implementation strategies were gained retrospectively, we were unable to formally capture their modifications over time. Future implementation efforts may be guided by the strategies outlined in Table 5, with tools such as the FRAME-IS used to document strategy modification in real time [54].

## Conclusion

Capitalizing on a unique opportunity, we explored factors influencing innovation implementation sequentially across the explanatory-to-pragmatic continuum. We have reported consistent, evolving and emerging factors that influence implementation related to the individual, innovation and inner setting. Our findings highlight the importance of iterative reviews of contextual factors across domains and along the implementation continuum. Specific to our example, findings indicate that higher-intensity walking protocols hold promise for widespread implementation in routine stroke rehabilitation. Rehabilitation teams were motivated to implement the protocol and found mechanisms to integrate this evidence-based practice within their unit amidst clinical realities. Despite innovation adaptations between trials, local modifications remained common as therapists aligned the protocol’s principles with their specific practice settings. This adaptability can facilitate implementation but may compromise fidelity. Derived from our relatively rare pragmatic trial, we identified key implementation strategies and operationalized these into practical actions from real-world implementation activities to offer tangible support for future implementation efforts. This approach may serve as a model for others to translate findings from their own implementation research. For long-term sustainability, evaluation of economic factors including staffing and resources requires careful consideration.

## Supplementary Information

Additional file 1: Trial design contextual factors_PRECIS-2 domains.pdf. Title. Table: Trial design contextual factors described using PRECIS-2 domain headings. Description: Trial design contextual factors described using PRECIS-2 domain headings for the explanatory Determining Optimal post-Stroke Exercise (DOSE) [9] and the more pragmatic Walk ‘n Watch (WnW) [13] stroke rehabilitation trials.

Additional file 2: COREQ Checklist.pdf. Title: COREQ-32 Checklist. Description: Completed Consolidated criteria for reporting qualitative research (COREQ): a 32-item checklist for interviews and focus groups.

Additional file 3: Walk ‘n Watch Interview guide.pdf. Title: Walk ‘n Watch Interview guide. Description: Staff interview guide using the Normalization Process Theory and Consolidated Framework for Implementation Research.

## Data Availability

The dataset supporting the conclusions of this paper is available in the Borealis Dataverse repository, [*unique persistent identifier and hyperlink to dataset(s) in http:// format – to be updated upon publication acceptance*]. Materials generated in this study are available from the corresponding author on request.

## Abbreviations

COREQ: COnsolidated criteria for REporting Qualitative research
CFIR: Consolidated Framework for Implementation Research
DOSE: Determining Optimal post-Stroke Exercise
ECG: Electrocardiogram
ERIC: Expert Recommendations for Implementing Change
HRR: Heart rate reserve
(M): Manager
NPT: Normalization Process Theory
PRECIS-2: PRagmatic Explanatory Continuum Indicator Summary-2
RCT: Randomized controlled trial
(T): Therapist
WnW: Walk ‘n Watch
6MWT: Six Minute Walk Test
