# Supplementary material for: Exploring factors influencing implementation across the explanatory-to-pragmatic trial continuum: a sequential qualitative integration of delivering higher-intensity walking exercise within inpatient stroke rehabilitation
Source: Implement Sci Commun. 2026 Jan 8;7:44. doi: 10.1186/s43058-025-00812-y (PMC12973730; doi:10.1186/s43058-025-00812-y)
Supplement: Supplementary file 1 — Additional file 1: Trial design contextual factors_PRECIS-2 domains.pdf. Title. Table: Trial design contextual factors described using PRECIS-2 domain headings. Description: Trial design contextual factors described using PRECIS-2 domain headings for the explanatory Determining Optimal post-Stroke Exercise (DOSE) [9] and the more pragmatic Walk ‘n Watch (WnW) [13] stroke rehabilitation trials. [file 43058_2025_812_MOESM1_ESM.pdf]

Additional file 1 | Table: Trial design contextual factors described using PRECIS-2 domain headings

	DOSE	Walk 'n Watch (WnW)
Domain	Description	Description
Eligibility criteria	*Inclusion criteria:*<br>• Admitted for inpatient rehabilitation for a stroke<br>• Within 10 weeks poststroke<br>• Adult age<br>• Able to walk 5 metres with max 1-person assist and assistive/orthotic device as required<br>• Able to understand and follow instructions<br>• Successful completion of a graded exercise stress test<br>• Over-ground walking speed <1.0 m/s<br>• Lower extremity hemiparesis (<4/5 manual muscle grade in at least one of the major lower extremity muscles)<br>• Prestroke disability <2 on the Modified Rankin Scale<br>*Exclusion criteria:*<br>• Enrolled in a drug or exercise rehabilitation study<br>• Prestroke health condition that included a gait disorder, another neurological condition (e.g. Parkinson's), serious medical or painful condition (e.g. active cancer)	*Inclusion criteria:*<br>• Admitted for inpatient rehabilitation for a stroke<br>• Within 12 weeks poststroke<br>• Adult age<br>• Able to walk 5 steps with max 1-person assist and assistive/orthotic device as required<br>• Able to understand and follow instructions<br>• Medically stable (e.g. stable cardiovascular condition, no active cancer)<br>*Exclusion Criteria*<br>• Enrolled in another rehabilitation study<br>• Another neurological condition<br>• Expected to receive less than 2 weeks of inpatient physical therapy
Recruitment path	• Research coordinator recruited eligible patients<br>• Total N=75 (Usual care=25, DOSE1=25, DOSE2=25)	• Research coordinator recruited eligible patients for outcome measures<br>• In phase 2, therapists encouraged to apply protocol with all eligible patients<br>• Total N for outcome measures=306 (Phase 1 (Usual care)=162, (Phase 2 (Implementation)=144)
Setting	• 6 sites across 3 Canadian provinces<br>• Inpatient rehabilitation units located in large urban cities of academic centres<br>• Same setting as usual care	• 12 sites across 7 Canadian provinces<br>• Inpatient rehabilitation units located in large and small cities that provided service to large regions, including rural catchments<br>• Same setting as usual care
Organisation innovation	• One experienced front-line physical therapist trained per site with 1 backfill<br>• When a patient was randomized to the DOSE protocol, the patient was moved to the trained therapist's caseload<br>• Training involved in-person site-visit with hands-on practice with heart rate and step counter<br>• Screening involved the research team consulting the chart for contraindications and completion of a graded exercise stress test with ECG, with consultation with physicians if needed<br>• Managers not directly involved, except to approve trial<br>• Trained therapists liaised directly with research team and were mentored by the research team<br>• Trained therapists provided with step counter (Fitbit One, Fitbit Inc., USA) and heart rate monitor (Alpha Mio watch, MioGlobal., Canada). Rehabilitation units typically had specialised equipment (e.g. Bodyweight support treadmill)	• All physical therapists, and rehabilitation assistants if applicable, trained<br>• In phase 2, therapists were encouraged to apply the protocol to all eligible patients even if they did not consent to the outcome measures<br>• Training involved video-conference with hands-on practice with heart rate and step counter. Training recorded for resource for new staff or as review for trained staff.<br>• Screening involved therapists consulting the chart for contraindications and completion of a 6MWT and resting blood pressure measurement, with consultation with physicians if needed<br>• Managers (practice leaders and supervisors) engaged for trial approval and to organize protocol training<br>• Some sites had practice leaders act as site coordinator, who were an implementation liaison between research team and therapists<br>• Rehabilitation unit provided with step counter (Fitbit Inspire, Alphabet Inc., USA) and heart rate monitor (Garmin Forerunner 235 and 735XT, Garmin Ltd., USA) for use with eligible patients. If consented to outcome measures, patients kept watch.

<table>
<tr><td>Flex of experimentation innovation – Delivery</td><td>• 3 trial arms: Usual care, DOSE1, DOSE2
• Usual care: Most trial sites had 60 mins (only 1 had 45 mins) sessions 5 d/wk
• DOSE1 innovation: Mandated to replace usual care activities for at least 30 mins, 5 d/wk, for 4 weeks. The therapist progressed the subject to (1) complete a minimum of 30 minutes at an intensity ≥40% HRR, gradually progressing to >60% HRR by the end of the 4 weeks; (2) achieve >2000 walking steps.
• DOSE2 innovation: In addition to DOSE1, therapists delivered a second daily session similar to DOSE1 innovation.
• Out of therapy session practice: Encouraged but no method to monitor</td><td>• 2 phases: Phase 1: Usual care, Phase 2: Implementation - protocol use replaces usual care for eligible patients
• Trial sites had 30 – 60 mins therapy sessions, 4 – 6 sessions weekly
• In phase 2 (Implementation): Advised protocol to be used for minimum 30 mins 5d/wk during inpatient stay but no strict mandate (2 sites 2x 30 mins/d for 5 d/wk). Content same as DOSE, except walking steps were refined aligning to baseline 6MWT:
<table>
<tr><td>6MWT baseline</td><td>Wk 1 step goal</td><td>Wk 2 step goal</td><td>Wk 4 step goal</td></tr>
<tr><td><100 m</td><td>1000</td><td>1500</td><td>2000</td></tr>
<tr><td>100-200 m</td><td>2000</td><td>2500</td><td>3000</td></tr>
<tr><td>>200</td><td>3000</td><td>3500</td><td>4000</td></tr>
</table>
• Out of therapy session practice: If deemed safe for independent walking, encouraged match steps outside of therapy (monitored with own watch if they have or number of minutes walked). If walking not deemed safe, focus on exercise to increase heart rate, monitored with own watch if they have or “talk test”/”rating of perceived exertion”.</td></tr>
<tr><td>Flex of experimentation innovation – Adherence</td><td>• Research team took responsibility of checking in with therapist at the front end of the trial, and for any new therapist, reviewing their intensity data and providing guidance
• Research team provided onboarding training and guidance to additional therapists if the original therapist left</td><td>• Rehabilitation unit was responsible for adherence to the protocol (e.g., short weekly huddles to discuss barriers and facilitators, review of intensity data)
• Rehabilitation unit responsible for onboarding new therapists to the protocol
• Support from research team for trial implementation only. No strict requirements were in place to adhere to the WnW protocol from the research team.</td></tr>
<tr><td>Follow up</td><td>• Physical measurements were largely part of usual care (walking speed, 6MWT, balance). Self-reported measurements were not always usual care.
• Follow-up timepoints were not all standard of care; would usually only have admission and discharge
• Timepoints: Admission, Discharge, 6-months, 12-months poststroke</td><td>• Physical measurements were largely part of usual care (walking speed, 6MWT, balance). Self-reported measurements were not always usual care.
• Follow-up timepoints were not all standard of care; would usually only have admission and discharge
• Timepoints: Admission, Discharge, 6-months, 9-months, 12-months poststroke</td></tr>
<tr><td>Primary outcome</td><td>• 6MWT at discharge / 4-weeks</td><td>• 6MWT at discharge / 4-weeks</td></tr>
<tr><td>Primary analysis</td><td>• Control (Usual care)=24 (n=1 was determined to not have a stroke and excluded from the analysis)
• DOSE1=25
• DOSE2=24 (n=1 did not complete innovation and excluded from the analysis)</td><td>• Phase 1 (Usual care)=149 (n=13 missing discharge assessment)
• Phase 2 (Implementation)=133 (n=11 missing discharge assessment)
• Intention to treat and per protocol design
• Multiple imputation methodology conducted for missing discharge data</td></tr>
</table>
