# Supplementary material for: Exploring factors influencing implementation across the explanatory-to-pragmatic trial continuum: a sequential qualitative integration of delivering higher-intensity walking exercise within inpatient stroke rehabilitation
Source: Implement Sci Commun. 2026 Jan 8;7:44. doi: 10.1186/s43058-025-00812-y (PMC12973730; doi:10.1186/s43058-025-00812-y)
Supplement: Supplementary file 2 — Additional file 2: COREQ Checklist.pdf. Title: COREQ-32 Checklist. Description: Completed Consolidated criteria for reporting qualitative research (COREQ): a 32-item checklist for interviews and focus groups. [file 43058_2025_812_MOESM2_ESM.pdf]

Additional file 2 | COREQ-32 Checklist (Tong et al. 2007)

No Item	Response
**Domain 1: Research**	
Personal Characteristics	
1. Interviewer / facilitator	Sarah Park (SSP) / Béatrice Ouellet (BO)
2. Credentials	Master of Rehabilitation Science / PhD student
3. Occupation	Research Assistant / Occupational Therapist
4. Gender	Female / Female
5. Experience and training	3 years' experience qualitative research / 9 years' experience conducting qualitative research in rehabilitation
Relationship with participants	
6. Relationship established	No
7. Participant knowledge of the interviewer	Participants were aware that the interviewer was not a direct part of the WnW trial management team, and that an honest perspective was wanted to learn lessons for implementation, with criticisms welcome.
8. Interviewer characteristics	Personal characteristics (as above) included in the 'Researcher characteristics and reflexivity' section.
**Domain 2: Study design**	
Theoretical framework	
9. Methodological orientation and theory	Framework analysis using the Consolidated Framework for Implementation Research (CFIR). Interview guide developed using Normalization Process Theory and CFIR.
Participant selection	
10. Sampling	Purposive. Therapist and rehabilitation assistants who had greater than two weeks of experience delivering the Walk 'n Watch (WnW) protocol to more than one patient during the WnW trial (Peters et al. 2025), and managers of the involved rehabilitation units, were invited to participate in one semi-structured interview. Potential therapists and rehabilitation assis-tants were staff who participated in a previous survey study regarding the implementation of the WnW protocol (Hung et al. 2025), and unit managers, who agreed to be contacted for future related research.
11. Method of approach	Email via managers
12. Sample size	14 therapists; 4 managers
13. Non-participation	Thirty-two staff (28 of 47 who completed the WnW survey study, and 4 unit managers) were invited to participate in the current study in May 2023. No participants dropped out.
Setting	
14. Setting of data collection	Online via Zoom
15. Presence of non-participants	No
16. Description of sample	WnW interviewees were all registered physical therapists and female. There was no rehabilitation assistant or male perspective garnered, although invited. Half (50%) the interviewees in this WnW qualitative study were aged between 30 – 39 yrs (all between 25 to 54). The majority (56%) had a Master's level of qualification, with the remaining having a Bachelor's degree. Manager interviewees had slightly more years of experience as health professionals than therapists (Managers: 20 ± 9, Therapists: 16 ± 8), as well as years of experience working with stroke patients (Managers: 13 ± 4, Therapists: 10 ± 8).
Data collection	

17. Interview guide	Interview guide was informed using the Normalization Process Theory and CFIR, and base on the guide used for the DOSE trial (Klassen et al. 2020). The interview guide was reviewed, revised and piloted by SP and BO. A French version of the interview guide was translated by a bilingual French native speaker (BO) and verified by a bilingual English native speaker (KB).
18. Repeat interviews	No
19. Audio/visual recording	Audio recording and verbatim transcription
20. Field notes	Field notes were taken as part of the WnW trial
21. Duration	30 – 60 minutes
22. Data saturation	No
23. Transcripts returned	No
**Domain 3: Analysis and findings**	
Data analysis	
24. Number of coders	Six (SA, SHH, LS, SJD, PT, LC). Each transcript was coded independently by two coders (SA and LC completing half each with a second coder), except the one which was coded independently by all coders to establish a shared understanding and interpretation of the coding framework.
25. Descriptions of the coding tree	Yes, coding framework based on the CFIR domains and constructs, with additional free codes developed where needed.
26. Derivation of themes	Pre-defined themes from the DOSE qualitative studies (Connell et al. 2018, Janssen et al. 2020), with additional emerging themes identified.
27. Software	NVivo 14 (Lumivero, USA)
28. Participant checking	No
Reporting	
29. Quotations presented	Illustrative quotes provided Tables 1 - 4
30. Data and findings consistent	Yes
31. Clarity of major themes	Provided in Tables 1- 4, and in interview result section text
32. Clarity of minor themes	Included in Table 1 – 4

References:

Connell LA, Klassen TK, Janssen J, Thetford C, Eng JJ. Delivering intensive rehabilitation in stroke: factors influencing implementation. Physical therapy. 2018;98(4):243-50.

Hung SH, Ackerley S, Connell LA, Bayley MT, Best KL, Donkers SJ, et al. Real-World Experiences of Therapy Staff Implementing an Intensive Rehabilitation Protocol in Canadian Stroke Inpatient Rehabilitation Settings: A Multi-Site Survey Study. Physical Therapy. 2025:pzaf111.

Janssen J, Klassen TD, Connell LA, Eng JJ. Factors influencing the delivery of intensive rehabilitation in stroke: patient perceptions versus rehabilitation therapist perceptions. Physical therapy. 2020;100(2):307-16.

Klassen TD, Dukelow SP, Bayley MT, Benavente O, Hill MD, Krassioukov A, et al. Higher doses improve walking recovery during stroke inpatient rehabilitation. Stroke. 2020;51(9):2639-48.

Peters S, Hung SH, Bayley MT, Best KL, Connell LA, Donkers SJ, et al. Safety and effectiveness of the Walk 'n Watch structured, progressive exercise protocol delivered by physical therapists for inpatient stroke rehabilitation in Canada: a phase 3, multisite, pragmatic, stepped-wedge, cluster-randomised controlled trial. The Lancet Neurology. 2025;24(8):643-55.

Tong A, Sainsbury P, Craig J. Consolidated criteria for reporting qualitative research (COREQ): a 32-item checklist for interviews and focus groups. International journal for quality in health care. 2007 Dec 1;19(6):349-57.
