# Supplementary material for: Exploring factors influencing implementation across the explanatory-to-pragmatic trial continuum: a sequential qualitative integration of delivering higher-intensity walking exercise within inpatient stroke rehabilitation
Source: Implement Sci Commun. 2026 Jan 8;7:44. doi: 10.1186/s43058-025-00812-y (PMC12973730; doi:10.1186/s43058-025-00812-y)
Supplement: Supplementary file 3 — Additional file 3: Walk ‘n Watch Interview guide.pdf. Title: Walk ‘n Watch Interview guide. Description: Staff interview guide using the Normalization Process Theory and Consolidated Framework for Implementation Research. [file 43058_2025_812_MOESM3_ESM.pdf]

Additional file 3 | Walk 'n Watch Interview guide

Staff interview guide using the Normalization Process Theory and Consolidated Framework for Implementation Research (English version)

INTRODUCTION

1. We are collecting some basic demographic information so we can characterize our sample.
   a. Can you tell me your highest educational degree or certificate? (e.g., high school, Bachelor's degree, Master's degree, Doctoral/PhD, other).
   b. Can you tell me what age category you fall in within a 5-year window: e.g., 25-29; 30-34; 35-39; 40-44, etc.
   c. Can you tell me what gender (man, woman, non-binary, other) you identify with?
   d. Can you tell me how many years you have worked as a healthcare provider?
      a. How many years you have worked as a healthcare provider with stroke patients?
2. Can you describe your role in stroke rehabilitation?

CHARACTERISTICS OF THE INTERVENTION

1. Can you describe in your own words what the Walk 'n Watch study is investigating?
2. Can you describe in your own words what the Walk 'n Watch protocol is?
   a. How incorporated into usual care?
3. What are the essential components/characteristics of this protocol?

CHARACTERISTICS OF THE INDIVIDUALS

1. What is your opinion on intensive exercise programs (like the Walk 'n Watch protocol) for people with stroke?
   a. Benefits and limitations
2. How do you feel about using the Walk 'n Watch protocol in your clinical setting? *Optional question for exploration if needed:*
   a. Concerns regarding the use of the protocol?
   b. Confidence in using the protocol?
   c. From what you think/know, do you think your colleagues share the same feelings as you?

TRAINING (skip if not relevant)

1. Can you describe the training you received to use the Walk 'n Watch protocol?
   a. Were you a part of the original training, or were you onboarded afterwards?
2. What do you think about the training you received?
   a. What did you like?
   b. How could it be improved?
3. How do you feel when you completed the training?
   a. Was the training sufficient?
   b. Do you feel that you and your colleagues have the skills and knowledge to use the Walk 'n Watch protocol as it should be?

Managers / Leads / Supervisors / Coordinators

1. Can you describe what it was like to support your team to receive the WnW protocol training?
2. Can you describe what it was like to support your team to do the WnW protocol?
3. Can you describe the type of support your team needed to do the WnW protocol?

4. Can you describe the feedback/comments from your team when they doing the WnW protocol? This includes elements that worked well and elements that did not work well.

COHERENCE (Understanding and thoughts about Walk 'n' Watch before starting)

1. What do you think about the Walk 'n Watch protocol before using it?
   a. From what you think/know, what did the clinicians on your team think?
2. What do you think about the protocol after using it?
   a. From what you think/know, what do the clinicians on your team think?

3. How is the Walk 'n Watch protocol different from what therapists treating stroke usually do?
4. From what you think/know, what do the other staff members (e.g., clinicians in other disciplines, and managers) think of the Walk 'n Watch protocol?
   a. What did they like and dislike?
   b. Do staff members think the Walk 'n Watch protocol is worth the effort?
5. From what you think/know, how did patients feel during Walk 'n Watch sessions?

a. Do you think the patients understand what they have to do in the Walk 'n Watch protocol?
b. Do you think the patients perceive the Walk 'n Watch protocol as worth the effort?

6. Based on your experience using the Walk 'n Watch protocol, what do you think about its fit into inpatient rehabilitation?
   a. Can it be used as it should be?
   b. What facilitates its use? What are the barriers to its use?
   c. How can we improve the protocol to make it easier to use?

COGNITIVE PARTICIPATION (Relational work people do to build and sustain a new complex intervention)

1. What instructions have you received in your setting regarding the use of the Walk 'n Watch protocol?
   a. How did you find these instructions?
   b. Was there enough direction in getting going at the start?
2. How did your social work environment (e.g., management, colleagues in your discipline/other disciplines) influence the use of the Walk 'n Watch protocol?
   a. Facilitators
   b. Barriers
3. Are other colleagues using the Walk 'n Watch protocol? Why?
4. How has the Walk 'n Watch protocol influenced the organization of your work?

a. Schedule
b. Duration of your intervention sessions
c. Work of your colleagues

COLLECTIVE ACTION (Operational work that people do to use the intervention)

1. **What opportunities have you had to use the Walk 'n Watch protocol with patients?**
   a. **What type of patients did you use it with?**
   b. **What criteria did you use to determine whether or not you could use the** Walk 'n Watch protocol with a client?

- What did you think of the safety assessment process?
- Criteria other than those proposed in the protocol? E.g., fatigue, pain, perceived assumptions and stereotypes, severity, cognition, etc.

c. With which other clinical activities did you combine the Walk 'n Watch protocol (task training, visual tasks, etc)?

d. How have you used the Walk 'n Watch protocol outside the scope of the study?

2. How did the implementation process of the Walk 'n Watch protocol go in your clinical setting?
    a. Facilitators and barriers to the implementation?

REFLEXIVE MONITORING (Reflection on the use and sustainability of the intervention)

1. How do you measure if the Walk 'n Watch protocol is working or not?
2. What do you think about the objectives proposed in the Walk 'n Watch protocol?
    a. What do you think about duration/ step count/ heart rate information?
    b. How have you used this information to progress patients?
3. What are your thoughts on using technology to measure objectives/patient progress (Garmin watch, Fitbit)? *Document ease of use, access, etc.*
    a. How can these technologies influence patient motivation?
4. If a patient is still on the unit after s/he finishes the Walk 'n Watch protocol, do you continue to use it during physical therapy?
    a. If not, what treatment do you replace it with?
5. Will you/ your team continue to use the Walk 'n Watch protocol in practice?
    a. What factors would influence this decision?
6. How has your involvement in the Walk 'n Watch study influenced your clinical practice with stroke patients?
    a. Influence on the practice of your team?
